# A prognostic nomogram based on competing endogenous RNA network for clear‐cell renal cell carcinoma

**DOI:** 10.1002/cam4.4109

**Published:** 2021-06-30

**Authors:** Yun Peng, Shangrong Wu, Zihan Xu, Dingkun Hou, Nan Li, Zheyu Zhang, Lili Wang, Haitao Wang

**Affiliations:** ^1^ Tianjin Institute of Urology The 2nd Hospital of Tianjin Medical University Tianjin China; ^2^ Department of Oncology Tianjin Medical University Second Hospital Hexi, Tianjin China

**Keywords:** competing endogenous RNA, gene signature, nomograms, renal cell carcinoma

## Abstract

**Background:**

Clear‐cell renal cell carcinoma (ccRCC) is stubborn to traditional chemotherapy and radiation treatment, which makes its clinical management a major challenge. Recently, we have made efforts in understanding the etiology of ccRCC. Increasing evidence revealed that the competing endogenous RNA (ceRNA) was involved in the development of varied tumors. However, a comprehensive analysis of the prognostic model based on lncRNA‐miRNA‐mRNA ceRNA regulatory network of ccRCC with large‐scale sample size and RNA‐sequencing expression data is still limited.

**Methods:**

RNA‐sequencing expression data were taken out from GTEx database and TCGA database, a total of 354 samples with ccRCC and 157 normal controlled samples were included in our study. The ccRCC‐specific genes were obtained by WGCNA and differential expression analysis. Following, the communication of mRNAs and lncRNAs with targeted miRNAs were predicted by MiRcode, starBase, miRTarBase, and TargetScan. A gene signature of eight genes was further constructed by univariate Cox regression, Lasso methods, and multivariate Cox regression analysis.

**Results:**

A total of 2191 mRNAs and 1377 lncRNAs was identified, and a dysregulated ceRNA network for ccRCC was established using 7 mRNAs, 363 lncRNAs, and 3 miRNAs. Further, a gene signature including eight genes based on this ceRNA was determined followed by the development of a nomogram predicting 1‐, 3‐, and 5‐year survival probability for ccRCC.

**Conclusion:**

It could contribute to a better understanding of ccRCC tumorigenesis mechanism and guide clinicians to make a more accurate treatment decision.

## Graphical abstract

We constructed a nomogram predicting 1‐, 3‐, and 5‐OS of ccRCC patients with a gene signature based on the ccRCC‐specific dysregulated ceRNA network and some other clinical factors. It could contribute to a better understanding of ccRCC tumorigenesis mechanism and guide clinicians to make a more accurate treatment decision.

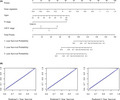

## 1 BACKGROUND

Kidney cancer, of which the most common subtype is clear‐cell renal cell carcinoma (ccRCC), constituted the third prevalent malignant tumor in the urogenital system of women and the second of men, and accounted for about 144,000 deaths annually worldwide.^1^ However, since a lack of external tumor factors such as age, nuclear grading, and microscopic tumor necrosis, it remains controversial for the optimum stratification of patients with ccRCC using the TNM staging system,^2^ hence, identification of prognostic predictive system for ccRCC containing both tumor anatomical features and other clinical and genetic variables deserves increasing attention.

Recently, the hypothesis of competing endogenous RNA (ceRNA) states that the pool of long non‐coding RNAs (lncRNAs) can regulate messenger RNAs (mRNAs) activity by binding to and competing for microRNAs (miRNAs).^3,4^ miRNA can regulate the expression level of targeted mRNAs with miRNA response elements (MREs) combining on the targeted mRNAs, on the other hand, lncRNA can serve as a molecular sponge to interact with miRNAs, which thus results in different kinds of human diseases process.^5^ At present, emerging evidence showed that the ceRNA hypothesis was involved in the development of different kinds of tumors, such as gastric, colon, liver, breast, bladder, and pancreatic cancer, which makes it possible to construct a prognostic prediction system on the basis of ceRNA network. Nevertheless, there are limited prognosis‐related ceRNA researches conducted in ccRCC. In this way, this study pointed to explore the prognostic significance of genes contained in the ccRCC‐specific dysregulated ceRNA network.

In the current study, ccRCC‐specific genes were obtained by employing weighted correlation network analysis (WGCNA)^6^ and differential expression analysis to RNA‐Seq data from Genotype‐Tissue Expression (GTEx)^7^ and The Cancer Genome Atlas (TCGA),^8^ then miRNA database was used to predict the interaction between mRNAs or lncRNAs and miRNAs. Following, 7 mRNAs, 363 lncRNAs, and 3 miRNAs were used to develop a dysregulated‐ceRNA network for ccRCC. Further, a gene signature of one mRNA (*MPP5*) and seven lncRNAs (*WT1*‐*AS*, *AC114316*.*1*, *AC103719*.*1*, *AL162377*.*1*, *HS1BP3*‐*IT1*, *LINC02657*, and *AC015909*.*1*) was constructed by univariate Cox regression, Least absolute shrinkage and selection operator (Lasso) methods, and multivariate Cox regression analysis. Consequently, a prognostic nomogram assessment system predicting 1‐, 3‐, and 5‐year survival probability was constructed by including the gene signature and related clinical characteristics using a stepwise Cox regression for ccRCC.

## 2 METHODS

### 2.1 Gene expression and clinical data

The high‐throughput RNA sequencing data of 539 kidney samples with ccRCC and 72 normal controlled samples were obtained from TCGA data repository using TCGAbiolinks^9^ R package. miRNA sequencing data were also retrieved from TCGA.^8^ Samples with tumor purity below 0.6,^10^ Formalin‐fixed paraffin‐embedded (FFPE) tissue, and duplicate samples were excluded in downstream analysis. Meanwhile, a total of 85 normal kidney cortex samples were downloaded from GTEx^7^ (version V8). No further approval was required from the Ethics Committee as the data comes from the TCGA and GTEx database. lncRNAs and mRNAs were recognized by the Ensembl^11^ database (version GRCh38.98). lncRNAs and mRNAs that were not included in the database were excluded in this study. We mainly used the R program (version 3.6.1)^12^ for the analysis in our study.

### 2.2 ccRCC‐specific mRNAs and lncRNAs

WGCNA, which can determine genes most related to a sample trait by clustering highly correlated genes to several modules and combining modules with external traits, was used to identify co‐expression network in lncRNAs or mRNAs expression profiles.^6^ The biweight midcorrelation analysis^13^ was efficiently used to assess weighted coexpression relationship. Gene significance (GS), module significance (MS), and module membership (MM) were explained by biweight midcorrelation coefficients. Genes in modules with max MS were defined with ccRCC importance, moreover, we only incorporated modules with a significant biweight midcorrelation coefficient between GS and MM. In this study, we obtained mRNAs or lncRNAs most related to ccRCC patients using WGCNA. Differentially expressed genes between ccRCC and normal samples were identified by DESeq2 with a threshold of |log2 fold change| >1 and adjusted *p*‐value < 0.01. Further, to obtain ccRCC‐specific mRNAs and lncRNAs, we intersected genes most positively or negatively correlated with ccRCC in WGCNA with upregulated or downregulated genes in differential expression analysis, respectively.

### 2.3 Construction of a dysregulated ceRNA network

Interactions between lncRNAs and miRNAs were identified by MiRcode.^14,15^ The interactions between mRNAs and miRNAs were explored by StarBase (version 3.9),^16^, ^17^ TargetScan (version 7.2),^18^ and miRTarBase (version 8.0)^19^ databases, meanwhile, miRNA sequencing data from TCGA was employed to review the top 10% expressed miRNA, since the implementation of ceRNA function depends on abundant of miRNAs, we only included triple lncRNAs‐miRNAs‐mRNAs with miRNA in above miRNAs. We used Cytoscape (version 3.7.1) to depict the ceRNA network.

### 2.4 Establishment of prognostic gene signature

To construct a risk assessment gene signature, a whole of 344 TCGA cases whose follow‐up >30 days with all clinical characteristics were randomly divided into a discovery group and a validation group, which was used to identify the gene signature and validate the efficacy of the gene signature, respectively. Both mRNA and lncRNA in the ceRNA network were employed to univariable Cox proportional‐hazards model. Then, we selected genes meeting the statistical significance (*p*‐value <0.01) to conduct Lasso penalized Cox regression analysis, which allows for the variable selection by constraining the variable regression coefficients, even to zero, and for declining the risk of overfitting,^20^ thus finding a prognostic gene signature for patients with ccRCC based on the Lasso‐penalized Cox regression model coefficients (β) and gene expression levels (risk scores = β1*gene^1^ + β2*gene^2^ +…..+ βn*gene^n^). To assess the prognostic gene signature, we conducted a time‐dependent receiver operating characteristic (ROC) curve analysis and calculated Harrell's concordance index (C‐index) among discovery group, validation group, and the entire group separately.

### 2.5 Construction of prognostic nomogram

A prognostic nomogram predicting 1‐, 3‐, and 5‐ year survival probability for ccRCC patients in the entire group was constructed by applying prognostic gene signature and relevant clinical characteristics to a stepwise Cox proportional‐hazards model. Further, we tested the discrimination and the calibration of the nomogram by Harrell's concordance index (C‐index) analysis and the calibration curves analysis.

### 2.6 Validation of gene signature and prognostic nomogram

The Human Protein Atlas and GEO database were firstly searched to validate the differential expression of genes in the gene signature. Bootstrap, which could adjust overfitting and provide nearly unbiased estimates for model performance, was employed to validate the gene signature and prognostic nomogram.^21^, ^22^ Let C‐index(orig) indicated C‐index based on the original data and gene signature or nomogram. First, we generated a bootstrap sample from our original data with replacement, and we developed a model based on the same process constructing the gene signature or prognostic nomogram using the upper bootstrap sample, we calculated C‐index based on this model using the bootstrap sample (C‐index(training)) and original data (C‐index(test)), optimism denoted to C‐index(training)–C‐index(test) which was regarded as the evaluation of overfitting. The corrected C‐index was C‐index(orig) subtracted optimism.

### 2.7 Functional enrichment analysis

We only applied functional enrichment analysis to mRNA as mRNA is the main functional molecule in ceRNA network. The clusterProfiler^23^ package was used to investigate both biological process (BP) in Gene Ontology^24^, ^25^ (GO) and functional pathways in Kyoto Encyclopedia of Genes and Genomes^26^ (KEGG).

## 3 RESULTS

### 3.1 Data source and data preprocessing

**FIGURE 1:**
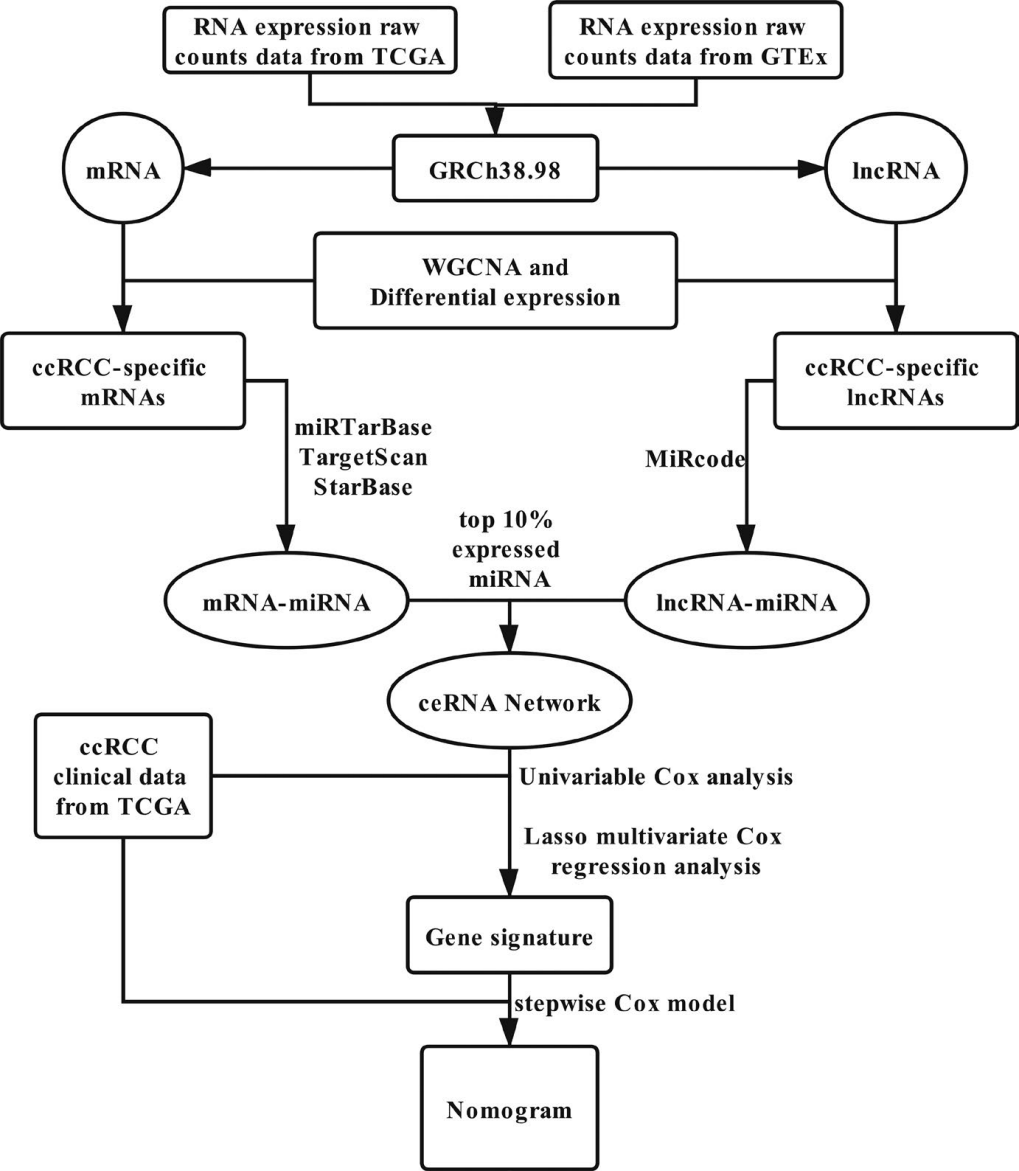
The flow chart of the ceRNA network construction

After filtering samples with tumor purity below 0.6,^10^ Formalin‐Fixed Paraffin‐Embedded (FFPE) tissue and duplicate samples, a total of 354 ccRCC samples and 72 normal controls in TCGA were included in downstream analysis. Meanwhile, a total of 85 normal controlled samples from GTEx was incorporated. Moreover, Ensembl identified 19538 mRNA expression values and 13511 lncRNA expression values for further analysis. The flow chart of the ceRNA network construction and the development of following gene signature and nomogram was depicted (Figure 1).

### 3.2 mRNA modules correlated with ccRCC

**FIGURE 2:**
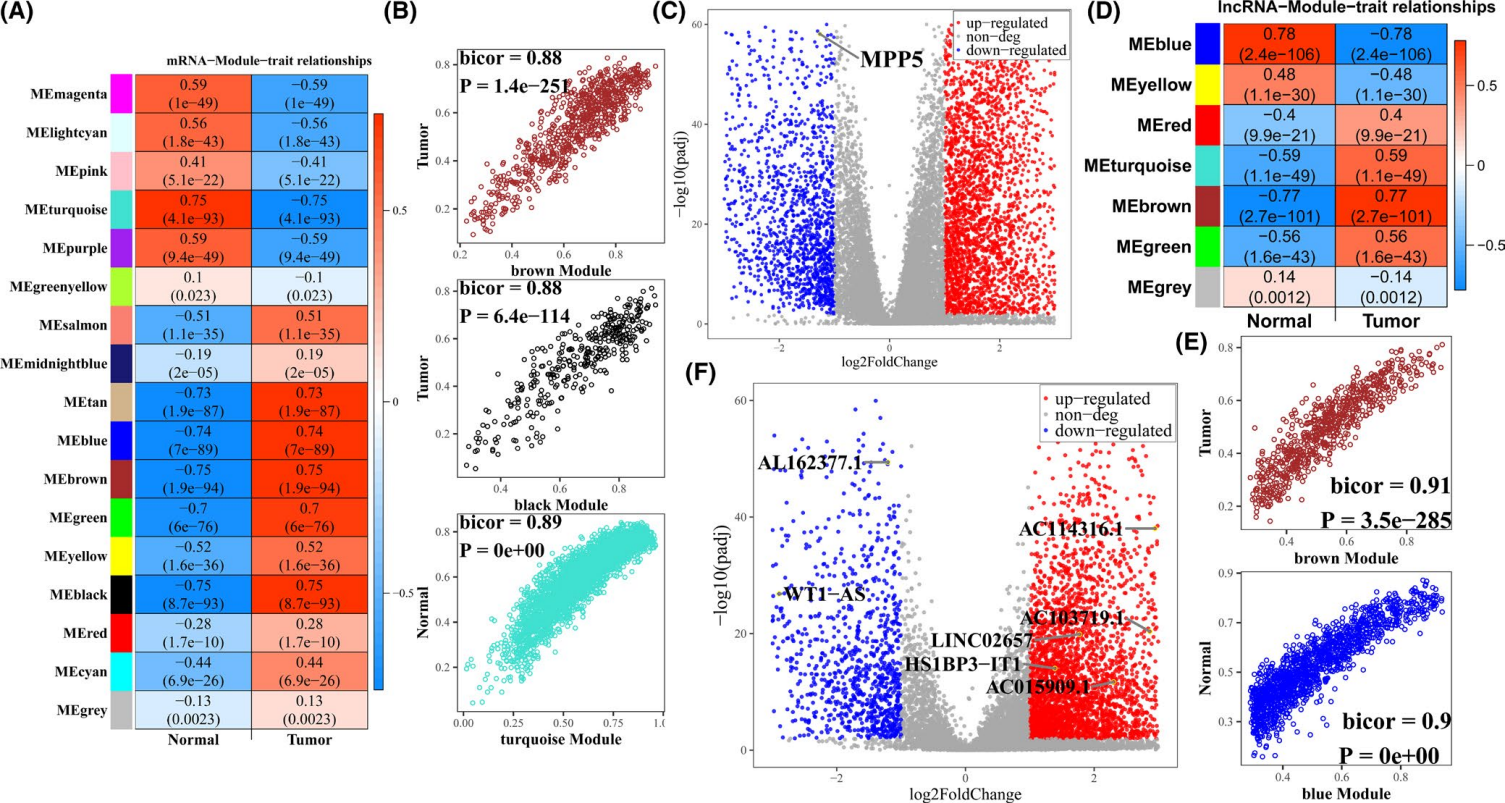
Determination of ccRCC‐specific mRNAs and lncRNAs. (A and D) the heatmap of the relationships between modules and traits was investigated, where the color represented the biweight midcorrelation coefficients; (A) mRNA (D) lncRNA. (B and E) Gene significance versus module membership. The x‐axis stands for the biweight midcorrelation coefficients between genes expression levels and the corresponding module eigengene, the y‐axis represents the biweight midcorrelation coefficients between genes expression levels with corresponding traits; (B) mRNA, (E) lncRNA. (C and F) the volcano of differential expression genes in ccRCC, red spots represent upregulated genes, and blue spots represent downregulated genes, further determined genes in prognostic gene signature were labeled and encircled in the yellow ring; (C) mRNA, (F) lncRNA

WGCNA was employed to analyze gene modules among the top 10000 mRNAs with maximum median absolute deviation (MAD) using softpower 14, minModuleSize 25, and mergeCutHeight 0.20 as the threshold. Consequently, we identified 17 gene color modules (Figures S1A–B). The biological process for each module was determined by GSEA (Table 1). As shown in Figure 2A, the association between gene co‐expression modules and ccRCC was explored, a total of 4005 mRNAs, which showed the highest relationship with ccRCC, was found in brown module, black module, and turquoise module. The relationships between MM and GS in three modules were then analyzed (Figure 2B). We then conducted GO analysis to reveal these mRNAs functions in BP, which found that these genes were most related to angiogenesis, extracellular matrix organization, and response to hypoxia (Figure S1C). Besides, genes were highly enriched in HIF‐1 signaling pathway, MAPK signaling pathway, and PI3K‐Akt signaling pathway by KEGG analysis (Figure S1D).

**TABLE 1:** Module description of mRNA modules created by WGCNA, GSEA was applied to disclose the biological process of modules. Module size indicated the number of genes in a module

Module	Module size	Biological process	*p*.adjust
Magenta	284	Adaptive immune response	7.02E‐26
Lightcyan	144	Nuclear‐transcribed mRNA catabolic process, nonsense‐mediated decay	1.13E‐39
Pink	310	Oxidative phosphorylation	4.06E‐38
Turquoise	2869	Adaptive immune response	1.42E‐32
Purple	263	Organic acid catabolic process	3.34E‐43
Greenyellow	236	Small molecule catabolic process	5.92E‐35
Salmon	213	Adaptive immune response	4.41E‐24
Midnightblue	151	Lymphocyte activation	1.40E‐57
Tan	214	Mitotic cell cycle process	1.48E‐35
Blue	1244	Lymphocyte activation	1.16E‐55
Brown	789	Lymphocyte activation	1.37E‐31
Green	397	Cotranslational protein targeting to membrane	4.49E‐56
Yellow	783	Sulfur compound metabolic process	1.12E‐17
Black	347	Adaptive immune response	8.34E‐19
Red	386	Vasculature development	3.11E‐40
Cyan	155	Organic acid catabolic process	2.18E‐34
Grey	1215	Mitotic cell cycle	5.44E‐19

### 3.3 Determination of differential expression mRNAs (DEmRNAs)

A total of 3679 significantly upregulated and 1944 significantly downregulated mRNAs, which were depicted in volcano map (Figure 2C), were identified by DESeq2. We used Gene Set Enrichment Analysis (GSEA) to demonstrate the biological function behind these identified differential expression genes. DEmRNAs were enriched in neutrophil‐mediated immunity, immune response‐activating signal transduction, and neutrophil activation in biological process (BP) (Figure S2A). Meanwhile, cytokine‐cytokine receptor interaction, human T‐cell leukemia virus 1 infection, and viral carcinogenesis‐related genes were found upregulated in DEmRNAs, while collecting duct acid secretion, proximal tubule bicarbonate reclamation, and glyoxylate and dicarboxylate metabolism pathways were downregulated in DEmRNAs (Figure S2B). Finally, we obtained 2191 ccRCC‐specific mRNAs by intersecting mRNAs from WGNCA modules and differential expression analysis.

### 3.4 ccRCC‐specific lncRNAs identified by WGCNA and DESeq2

We investigated the co‐expression network of 8332 lncRNAs by WGCNA with softpower 7, minModuleSize 15, and mergeCutHeight 0.20 as the threshold after filtering lncRNAs with median absolute deviation (MAD) zero. Finally, we identified a total of seven coexpression modules (Figures S3A–B) and found that brown module with 744 lncRNAs was most positively related to ccRCC and blue module including 1424 lncRNAs presented the highest negative relationship with ccRCC (Figure 2D and Table 2), Further, both modules possessed a significant relationship between MM and GS (Figure 2E). At the same time, we identified 3654 upregulated lncRNAs and 1223 downregulated lncRNAs (Figure 2F). By intersecting them with lncRNAs in brown and blue modules, respectively, we obtained 610 lncRNAs positively correlated with ccRCC, and 767 lncRNAs with a negative relation to ccRCC.

**TABLE 2:** The details of lncRNA modules created by WGCNA. Module Size indicated the number of genes in a module

Module	Blue	Brown	Green	Grey	Red	Turquoise	Yellow
Module size	1424	744	125	3014	92	2806	127

### 3.5 Development of dysregulated ceRNA network

**FIGURE 3:**
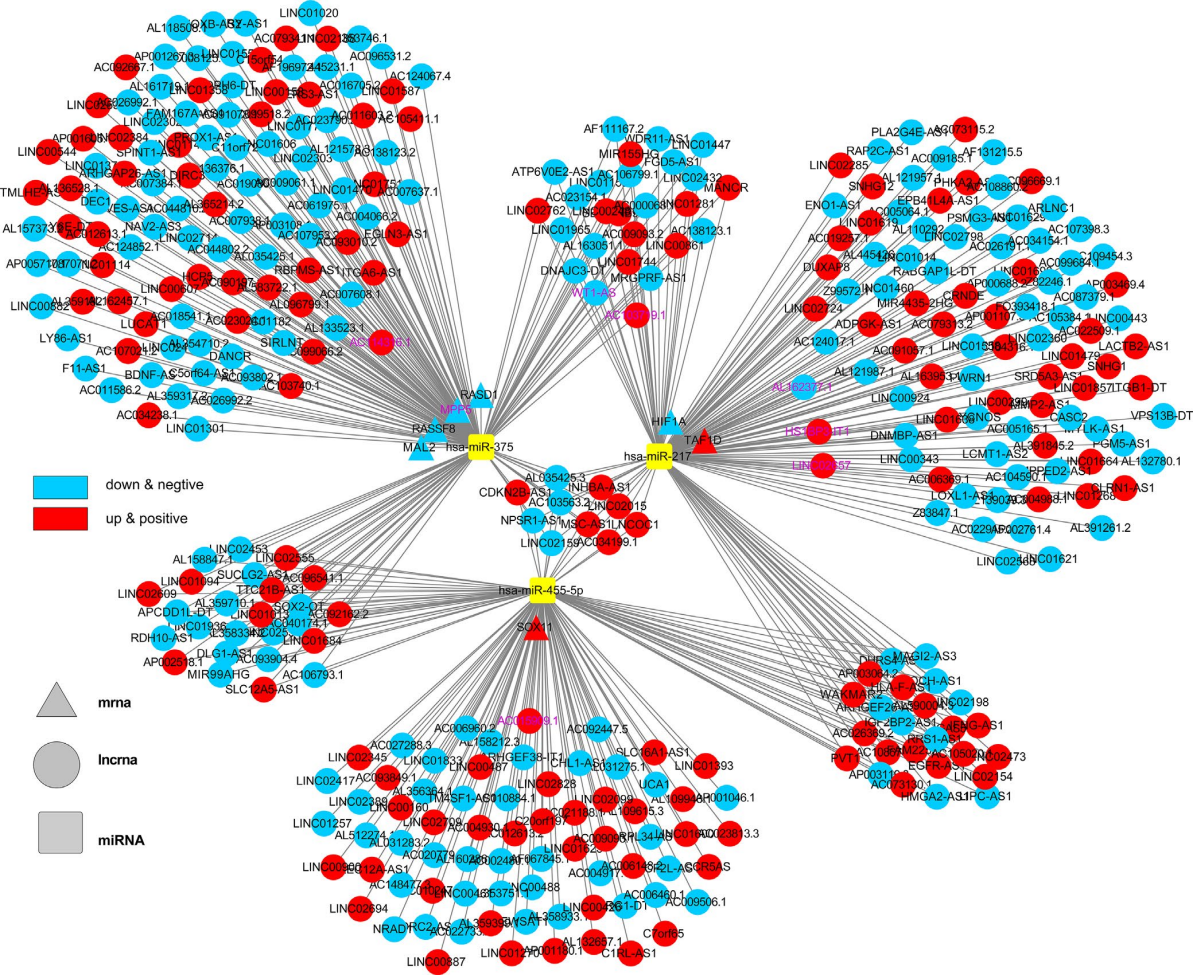
A lncRNA‐miRNA‐mRNA ceRNA network was constructed by 363 lncRNAs, 3 miRNAs, and 7 mRNAs. Further determined genes in prognostic gene signature were labeled in purple color

A total of 2191 ccRCC‐specific mRNAs and 1377 ccRCC‐specific lncRNAs was included in the construction of dysregulated ceRNA network. Then the interaction between lncRNAs and miRNAs was identified using miRcode, following StarBase, miRTarBase, and TargetScan databases were applied to demonstrate the targeted miRNA of cancer‐specific mRNAs. At the same time, miRNA sequencing data from TCGA was employed to review the top 10% expressed miRNA, we only included triple lncRNAs‐miRNAs‐mRNAs with miRNA in the above miRNAs. Consequently, a dysregulated ceRNA network for ccRCC was established using 7 mRNAs, 363 lncRNAs, and 3 miRNAs (Figure 3).

### 3.6 Construction of gene signature using genes in the ceRNA network

**FIGURE 4:**
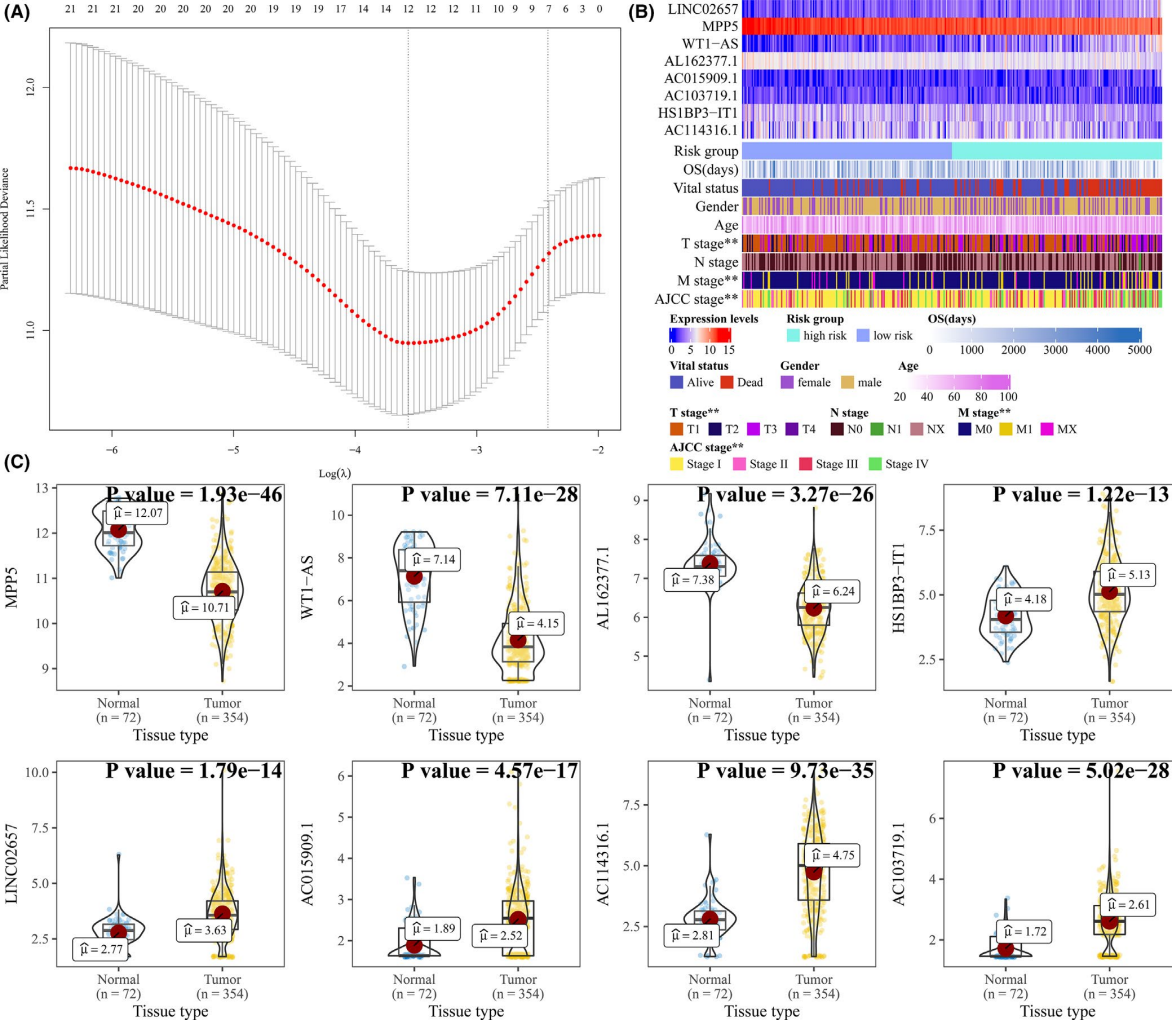
A gene signature based on ccRCC‐specific ceRNA network was developed. (A) Coefficient paths for Lasso regression model in dependence on log(λ). (B) The expression levels of eight genes in gene signature associated with clinical characteristics were also depicted in a heatmap. The risk group of high risk and low risk was based on median risk scores. Categorical variables were tested by Chi‐squared and continuous variables were tested by analysis of variance, label (*) means *p *< 0.05, label (**) means *p *< 0.01, and label (***) means *p *< 0.001. (C) The differential expression of the eight genes in the gene signature was compared

**FIGURE 5:**
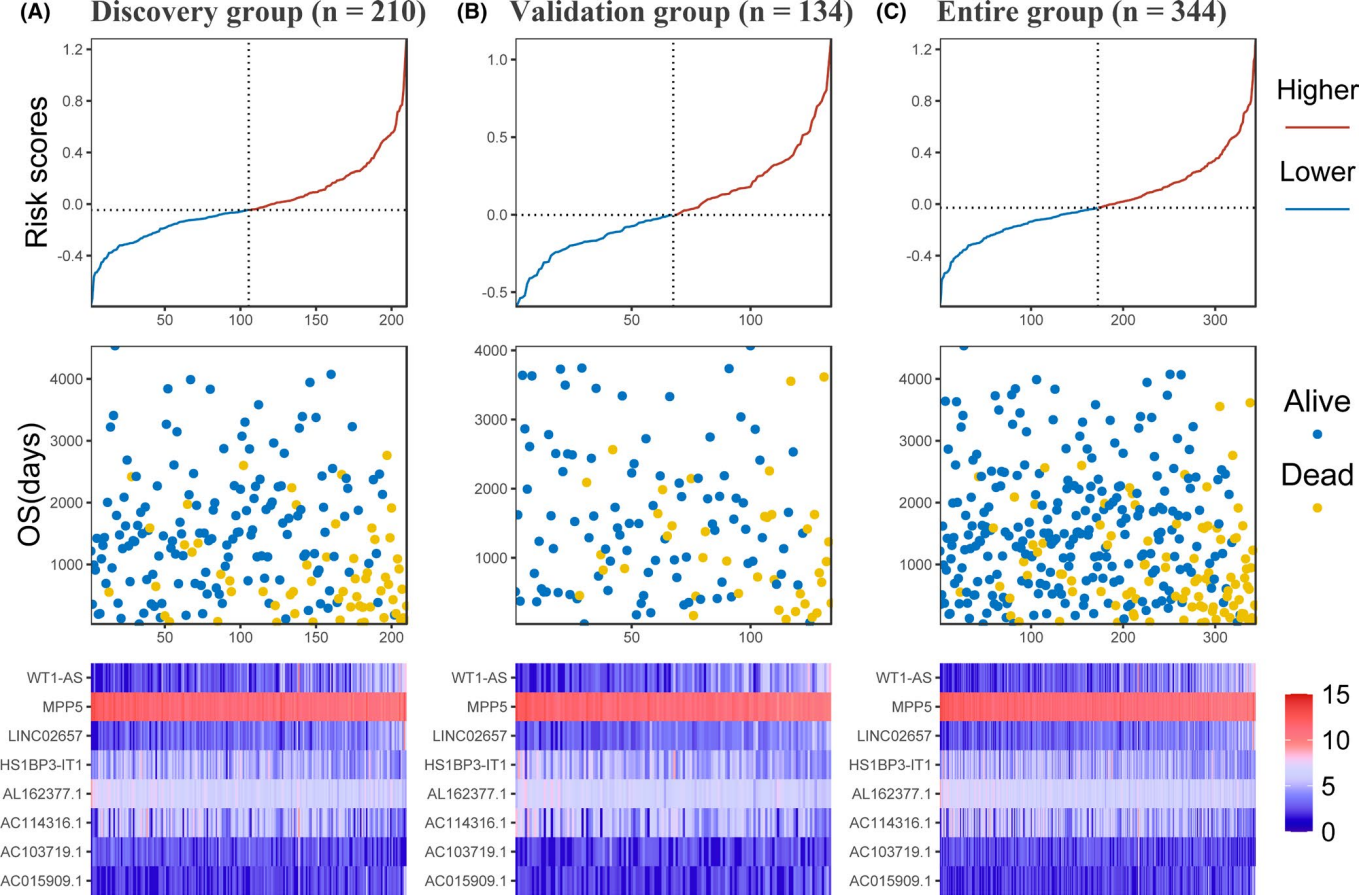
Analysis of gene signature risk score in ccRCC patients. (A) Discovery group (B) validation group (C) entire group. The samples ranked according to risk scores correspond to x‐axis. Each panel consists of three rows: top row, y‐axis represents risk scores, and the risk group of higher and lower was based on median risk scores; middle row, y‐axis represents overall survival time in days; bottom row, heatmap showing the expression of the eight key genes. The color from blue to red shows the expression levels from lower to higher

A total of 344 ccRCC patients with the expression levels of genes in the dysregulated ceRNA network were included in the construction of the gene signature. We randomly classified patients into two groups: a discovery group (*n* = 210) and a validation group (*n* = 134). Meanwhile, the univariate Cox proportional hazards model was applied to both lncRNAs and mRNAs in the dysregulated ceRNA network to screen for genes as biomarkers which significantly influence overall survival and prognosis in discovery group. Consequently, we obtained 21 genes including 1 mRNA and 20 lncRNAs with the threshold of *p*‐value <0.01, subsequently these genes were applied into Lasso with a lambda based on Cross‐Validation (Figure 4A) using glmnet.^27^ Finally, we obtained eight genes, namely *MPP5*, *WT1*‐*AS*, *AC114316*.*1*, *AC103719*.*1*, *AL162377*.*1*, *HS1BP3*‐*IT1*, *LINC02657*, *and* *AC015909*.*1*. The expression level of these genes associated with clinical characteristics was also depicted in a heatmap (Figure 4B). Furthermore, we also compared the expression levels between ccRCC and normal control samples (Figure 4C). A risk score was constructed based on Lasso Cox model coefficients and the gene expression levels (risk score = 0.102*LINC02657–0.183*MPP5 + 0.0979*WT1‐AS–0.111*AL162377.1 + 0.000866*AC015909.1–0.0372*AC103719.1–0.0357*HS1BP3‐IT1–0.0352*AC114316.1). The risk scores, overall survival time, and expression levels of model genes was explored in Figure 5A–C, and the clinical characteristics between groups determined by median risk scores were shown in Table 3.

**TABLE 3:** Relationship between clinicopathological characteristics and risk score calculated by using the eight‐gene signature

	Level	Low risk	High risk	*p*
N		172	172	
Vital status (%)	Alive	151 (87.8)	93 (54.1)	<0.001
	Dead	21 (12.2)	79 (45.9)	
OS (mean [SD])		1515.10 (1008.39)	1308.04 (971.94)	0.053
Gender (%)	Female	57 (33.1)	68 (39.5)	0.262
	Male	115 (66.9)	104 (60.5)	
Ages (mean [SD])		60.40 (12.48)	60.88 (11.64)	0.711
T stage (%)	T1	114 (66.3)	81 (47.1)	0.005
	T2	20 (11.6)	29 (16.9)	
	T3	36 (20.9)	58 (33.7)	
	T4	2 (1.2)	4 (2.3)	
N stage (%)	N0&NX	172 (100.0)	168 (97.7)	0.131
	N1	0 (0.0)	4 (2.3)	
M stage (%)	M0&MX	158 (91.9)	139 (80.8)	0.005
	M1	14 (8.1)	33 (19.2)	
AJCC stage (%)	Stage I	114 (66.3)	79 (45.9)	0.001
	Stage II	18 (10.5)	21 (12.2)	
	Stage III	25 (14.5)	36 (20.9)	
	Stage IV	15 (8.7)	36 (20.9)	
Gene signature (mean [SD])		−0.21 (0.15)	0.24 (0.25)	<0.001

The group of low risk and high risk was based on a cut point of median risk scores. Categorical variables were tested by Chi‐squared and continuous variables were tested by analysis of variance.

### 3.7 Estimation and validation of gene signature

**FIGURE 6:**
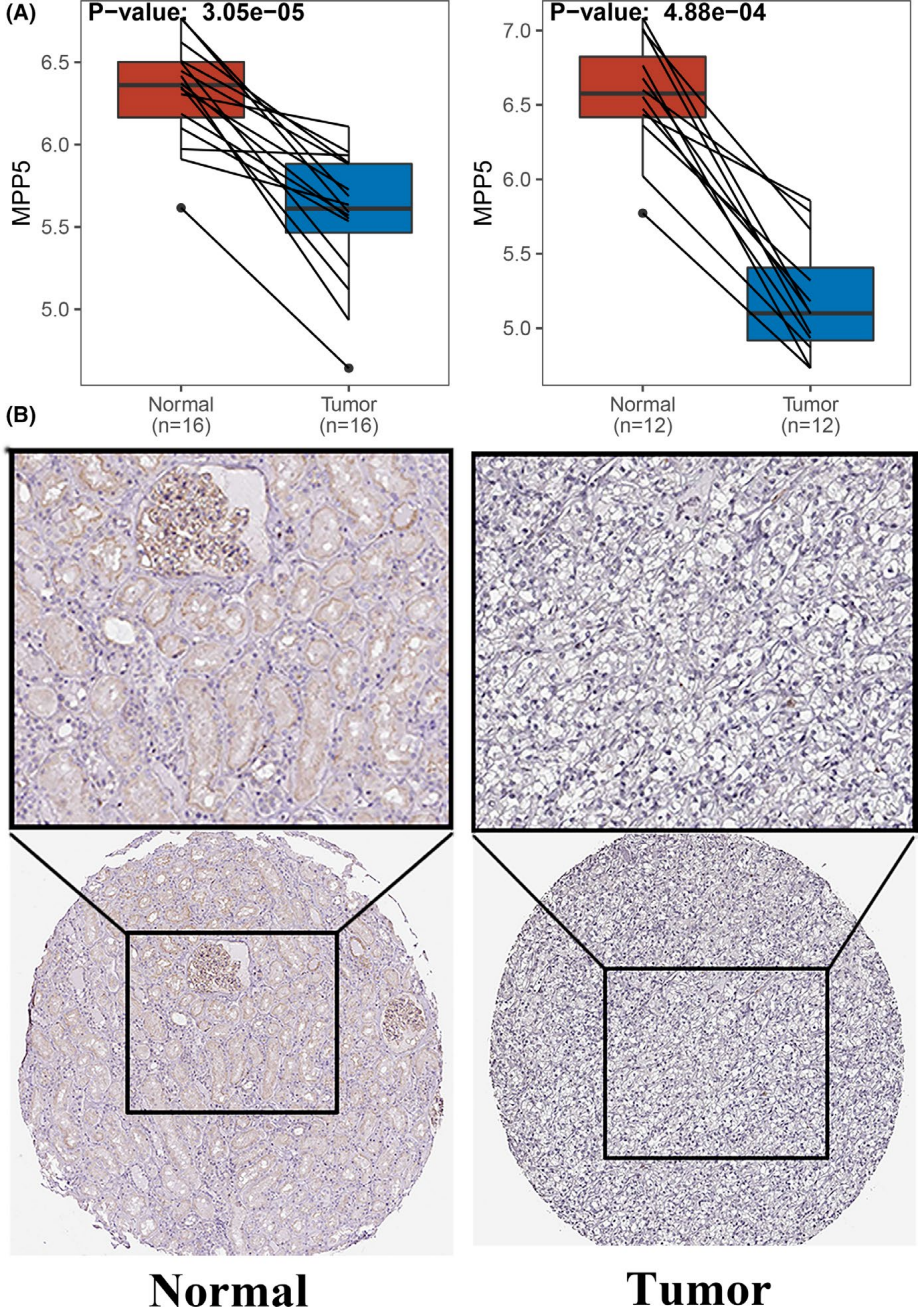
The expression levels of MPP5 in GEO and The Human Protein Atlas. (A) Boxplot of MPP5 in GSE76207 (Left panel) and GSE82122 (Right panel). *p*‐value was calculated by paired Wilcoxon Rank Sum test. (B) Differential protein level of MPP5 based on the human protein atlas

**FIGURE 7:**
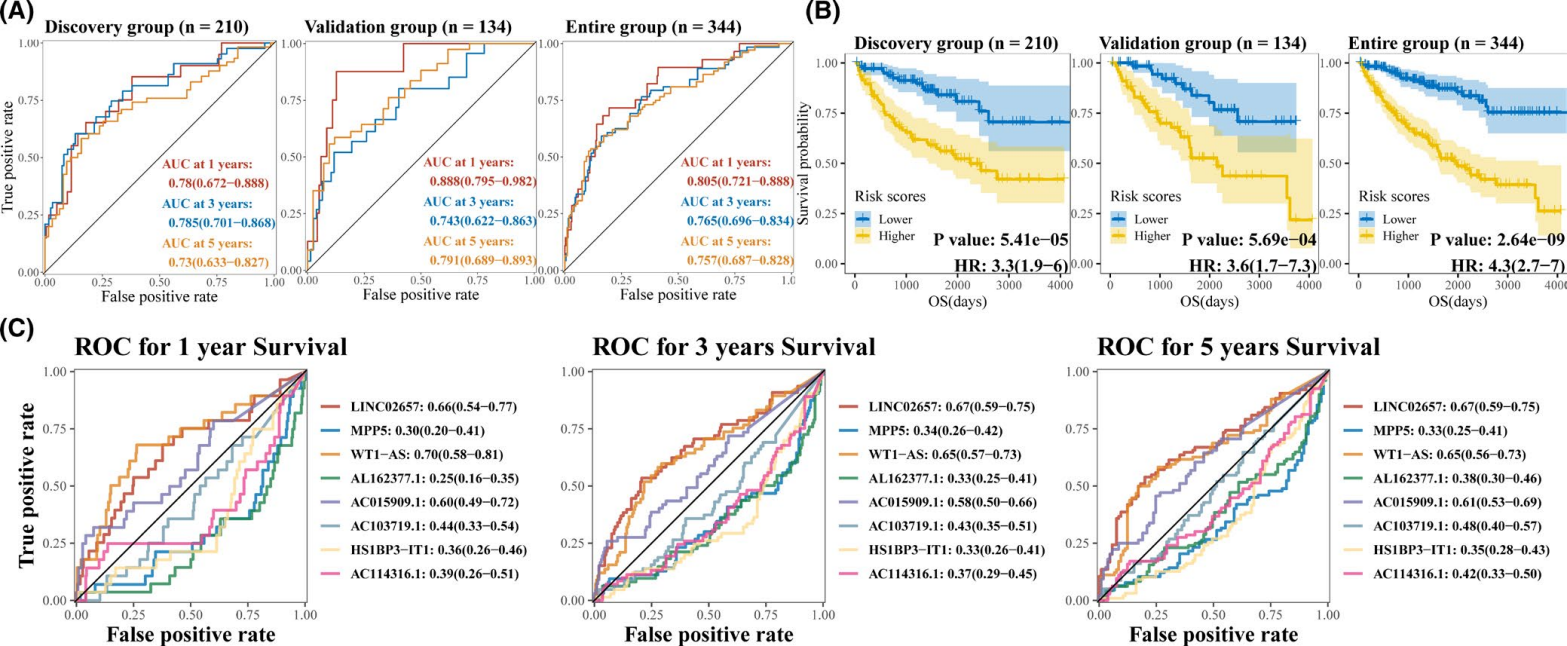
Estimation and validation of gene signature. (A)Time‐dependent ROC curve for predicting 1‐, 3‐, and 5‐year survival with gene signature in discovery group, validation group and entire group. AUC along with 95% CI was displayed. (B) Kaplan–Meier survival curves showing overall survival outcomes for the high‐ and low‐risk patients in discovery group, validation group and entire group, the risk group of high risk and low risk was based on median risk scores based on gene signature. (C) Time‐dependent ROC curve for predicting 1‐, 3‐, and 5‐year survival with genes in the gene signature was explored in the entire group

To validate gene signature, we searched expression levels of genes contributing to the risk scores in the GSE76207 (*p*‐value: 3.05e‐05) and GSE82122 (*p*‐value: 4.88e‐04) which indicated the significance of MPP5 to ccRCC (Figure 6A). Protein levels of MPP5 between renal cancer and normal control samples were also verified in The Human Protein Atlas (Figure 6B). Furthermore, we calculated the C‐index for the prognostic model in discovery group (0.741, 95% CI, 0.678–0.805), ROC revealed the areas under ROC (AUC) among 1‐, 3‐, and 5‐ year were 0.780 (95% CI: 0.672–0.888), 0.785 (95% CI: 0.701–0.868), and 0.730 (95% CI: 0.633–0.827), respectively (Figure 7A). Kaplan–Meier analysis in discovery group also presented statistical significance that patients with predicted high risk showed shorter OS than those with low risk (*p*‐value: 5.41e‐05, Figure 7B). ROC analysis was conducted in both validation group (AUC at 1‐, 3‐, and 5‐year: 0.888, 0.743, and 0.791) and entire group (AUC at 1‐, 3‐, and 5‐year: 0.805, 0.765, and 0.757) (Figure 7A). Following, Kaplan–Meier analysis was conducted in validation group and entire group which both suggested prognostic significance to overall survival (Figure 7B). Moreover, C‐index analysis in validation group and entire group presented 0.754 (95% CI, 0.677–0.831) and 0.742 (95% CI, 0.691–0.792), respectively and the corrected C‐index adjusted by bootstrap was 0.721 (Table 4), which further confirmed the accuracy of the gene signature. Finally, ROC analysis was used with reference to genes in prognostic model in entire group data, which presented that WT1‐AS (0.70, 95% CI: 0.58−0.81) could provide the best prediction of 1 year survival, LINC02657 performed best in 3‐year (0.67, 95% CI: 0.59−0.75) survival and 5‐year survival (0.67, 95% CI: 0.59−0.75) (Figure 7C).

**TABLE 4:** Validation of gene signature and nomogram based on bootstrap methods. C‐index(orig) indicated C‐index based on the original data for gene signature or nomogram

	C‐index(orig)	Training	Test	Optimism	Index.corrected
Gene signature	0.742	0.766	0.745	0.021	0.721
Nomogram	0.809	0.81	0.804	0.006	0.803

The column training represented C‐index based on bootstrap model and bootstrap sample. The column test represented C‐index based on bootstrap model and original data, optimism denoted to C‐index(training)–C‐index(test) which was regarded as the evaluation of overfitting. The corrected C‐index was C‐index(orig) subtracted optimism.

### 3.8 Building and validation of a prognostic nomogram

**FIGURE 8:**
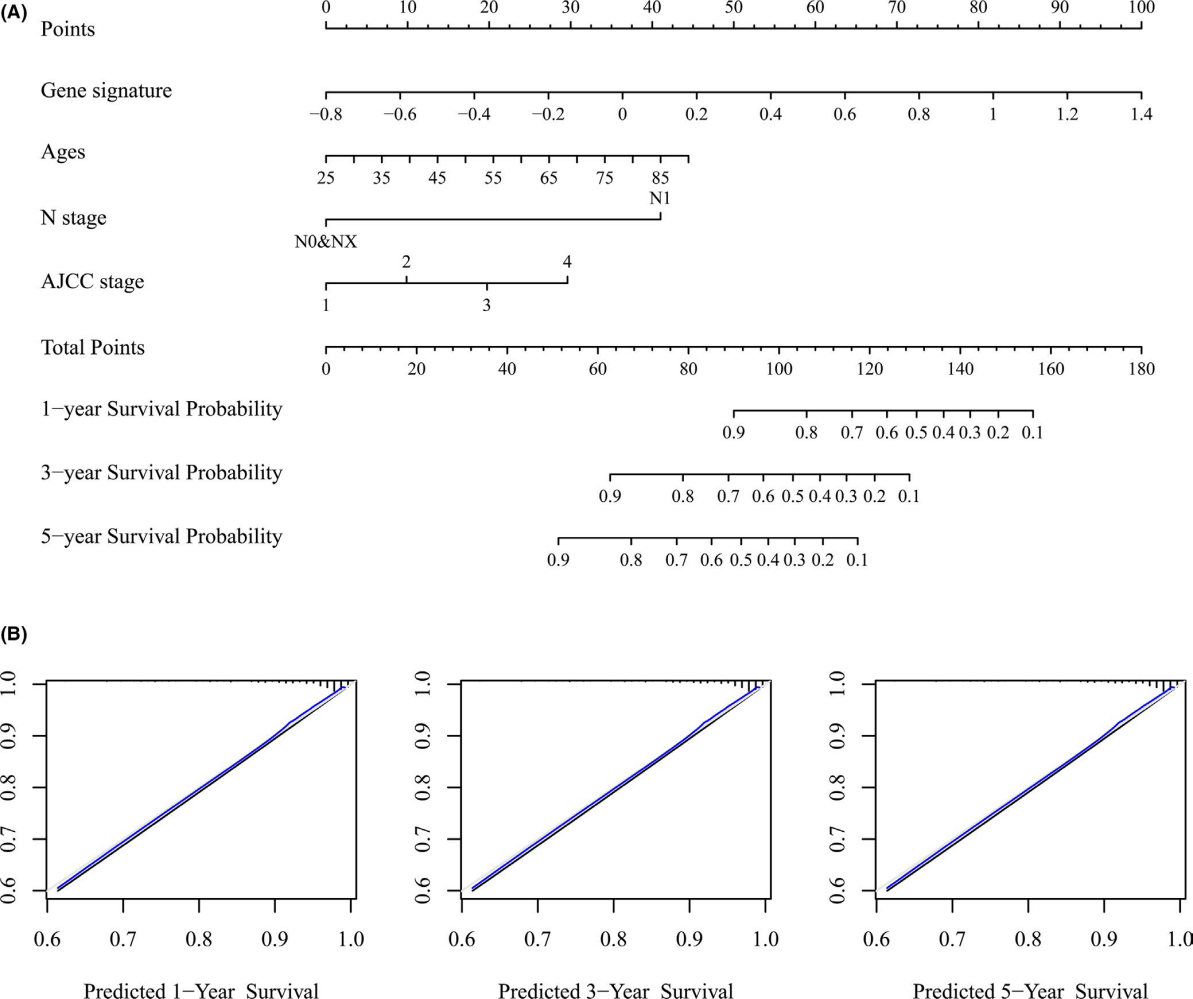
Building and validation of a prognostic nomogram. (A) Nomogram based on gene signatures, ages, N stage, and AJCC stage for 1‐, 3‐, and 5‐year OS prediction. The number from 1 to 4 in AJCC stage represents AJCC stage I, II, III, and IV, respectively (B) Calibration plot for agreement test between 1‐, 3‐, and 5‐year OS prediction and actual observation

Univariate Cox regression analysis showed that gene signature, ages, T stage, M stage, N stage, and AJCC stage were significantly related to the overall survival (*p* < 0.05), and multivariate Cox analysis further revealed that ages, N stage, and gene signature were independent risk factors. Meanwhile, a stepwise Cox regression model was employed to develop a nomogram predicting the 1‐, 3‐, and 5‐year OS for ccRCC patients (Figure 8A) with a C‐index 0.809 (95% CI, 0.696–0.887), we also depicted the calibration curve for 1‐, 3‐, and 5‐year survival probability (Figure 8B) which collectively indicated a good accuracy of the prognostic nomogram. Bootstrap validation further authenticated the performance of this nomogram (corrected C‐index: 0.803) (Table 4).

## 4 DISCUSSION

Kidney cancer accounted for 338,000 new cases and 144,000 deaths each year.^1^ The most common subtype of kidney cancer ccRCC, is a complex tumor with different clinical and pathological features, genetic variation, DNA methylation profiles, and RNA and proteomic signatures.^28^ Nevertheless, the TNM staging system, the most used risk assessment system for ccRCC patients, failed to consider these genomic variation of ccRCC which makes it not perfect for accurately predicting the prognosis of ccRCC patients.^29^

The novel hypothesis of gene expression regulation has been confirmed to be related to the mechanism of varied diseases, especially cancer. The disturbance of the equipoise of ceRNA network was of vital importance for tumorigenesis. In gallbladder cancer, the lncRNA *PVT1* which was positively related to malignancies and worse overall survival time was upregulated in gallbladder cells. *PVT1* and *HK2* act as a ceRNA of miR‐143, which could regulate aerobic glucose metabolism in gallbladder cancer cells, and promote cell proliferation and metastasis.^30^ *PTAR* acts as a ceRNA of miR‐101 which promotes tumorigenicity and metastasis of ovarian cancer in vivo.^31^ LncRNA *DANCR* functions as a ceRNA in osteosarcoma which could promote cell proliferation and metastasis.^32^ *MT1JP*, which severs as a ceRNA regulating *FBXW7* expression, could influence the progression of gastric cancer.^33^ Thus, ceRNA network containing crucial biomarkers was of vital importance in tumorigenesis. Importantly, lncRNA‐miRNA‐mRNA dysregulated ceRNA network played a vital role in predicting disease prognosis. For example, in pancreatic cancer, 11 lncRNAs have been found and validated to function well in predicting prognosis.^34^ Seven genes (*LPP*‐*AS2*, *MUC1*, *GAB2*, *hsa*‐*let*‐*7i*‐*5p*, *hsa*‐*let*‐*7f*‐*5p*, *hsa*‐*miR*‐*101*‐*3p*, *and* *hsa*‐*miR*‐*1226*‐*3p*) in a recurrent soft tissue sarcoma‐specific ceRNA network associated with recurrence and survival were identified based on the TCGA database.^35^ Although there were many studies on ceRNA networks conducted in numerous cancers, nevertheless, few of them were related to ccRCC.

In this study, ccRCC‐specific mRNAs and lncRNAs, including 2191 mRNAs and 1377 lncRNAs, were identified by WGCNA and DESeq2. Functional enrichment analysis revealed these mRNAs involved in MAPK signaling pathway and PI3K‐Akt signaling pathway both of which have been reported to tightly implicated in the progression and metastasis of malignancy.^36^, ^37^, ^38^, ^39^ Sunitinib and axitinib, the most common targeted drugs related to MAPK signaling pathway, were used extensively in renal cancer.^40^ GSEA suggested that these mRNAs were highly involved in multiply immune‐related function and pathways, like neutrophil‐mediated immunity, immune response‐activating signal transduction, and neutrophil activation in biological process and Cytokine‐cytokine receptor interaction and Human T‐cell leukemia virus 1 infection in KEGG, this may imply the importance to ccRCC of immune infiltration, which has been validated to play vital role in tumor growth and progression.^41^, ^42^ Importantly, axitinib plus immune checkpoint inhibitor has made great success in treating renal carcinoma.^43^, ^44^ Our findings may provide evidence for the combination of tyrosine kinase inhibitors and immunotherapy.

Subsequently, the dysregulated ceRNA network consisting of 363 lncRNAs, 3 miRNAs, and 7 mRNAs was determined by MiRcode, StarBase, miRTarBase, and TargetScan databases. To further explore the relationships with prognosis of these 370 genes (mRNA and lncRNA in the ceRNA network), a gene signature with eight genes, namely *MPP5*, *WT1*‐*AS*, *AC114316*.*1*, *AC103719*.*1*, *AL162377*.*1*, *HS1BP3*‐*IT1*, *LINC02657*, and *AC015909*.*1*, was determined by univariate Cox proportional hazard regression, Lasso and multivariate Cox proportional hazard regression analysis. The discriminations and accuracy of the gene signature were validated with C‐index and time‐dependent ROC curve, which all suggested that the eight genes in the model could act as biomarkers based on the patients’ prognosis. Moreover, bootstrap validations also authenticated the good performance of the gene signature.

Among the eight genes in the gene signature, the exclusive mRNA MPP5, which is associated with the membrane‐associated guanylate kinase family helping the construction of cell polarity, had been validated to be associated with the maintenance of cell polarity, invasion, and cell division in prostate cancer,^45^ meanwhile, disruption of apical protein *MPP5*, which could negatively regulate YAP/TAZ abundance and activity, might promote the enrichment of oncogenic YAP and TAZ in hepatocellular carcinoma.^46^ The loss of MPP5 is a hallmark of cancer is crucial for tissue organization, corresponded to the downregulated expression in ccRCC. Long noncoding RNA *WT1*‐*AS* which functioned as a potential tumor suppressor was related to poor survival in cervical squamous cell carcinoma^474849^ and triple‐negative breast cancer (TNBC).^50^ For lncRNA *HS1BP3*‐*IT1*, it may be a prognosis biomarker for cholangiocarcinoma,^51^ laryngeal cancer,^52^ respectively. *LINC02657* has been named as LASTR *(lncRNA* *associated with SART3 regulation of splicing)*, which was reported to decline the fitness of cancer cells by inducing intron retention.^53^ Therefore, our prediction of the ceRNA network had great confirmation of previous studies.

Nomograms were widely used as prognostic tools in oncology and medicine. By including various prognosis‐associated variables and generating survival probability, nomograms can help clinicians make better treatment decisions.^54^ In the present study, by including gene signature based on the dysregulated ceRNA network and other related clinical characteristics into a stepwise Cox model, a concise nomogram for the prognostic prediction of ccRCC was developed, meanwhile, C‐index 0.809 (95% CI, 0.696–0.887) and calibration curve (Figure 8B) all suggested its perfect discriminations and calibrations. Bootstrap validation (corrected C‐index: 0.803) (Table 4) for the nomogram further strengthened the effect in predicting the prognosis of ccRCC comparing with Jiang's nomogram (C‐index 0.79; 95% CI 0.75–0.82).^55^

However, there are still some limitations in our study. Although the prognostic dysregulated‐ceRNA‐related gene signature and nomogram were validated by internal dataset and bootstrap, the exact mechanism of the eight genes in the gene signature have not been explored in the current study and an externally validated dataset is necessary for both gene signature and nomogram. Therefore, further efforts to investigate the exact function of eight genes in ccRCC in vitro and in vivo and external validation based on a larger sample size are still required to make our findings more convincible, which is also the direction of our future work.

## 5 Conclusions

In conclusion, a ccRCC‐specific dysregulated ceRNA network was developed, followed by the determination of an eight‐gene signature, which will help us better understand dysregulated ceRNA network‐mediated pathway in ccRCC. Moreover, the development of nomogram, including both clinical characteristics and ccRCC‐specific gene signatures, could accurately predict 1‐, 3‐, and 5‐year OS of ccRCC, it could contribute to a better understanding of ccRCC tumorigenesis mechanism and guide clinicians to make a better treatment decision.

## CONFLICT OF INTEREST

None.

## Supporting information

Figure S1

Figure S2

Figure S3

## Data Availability

The datasets analyzed for this study can be found in the GTEx (https://www.gtexportal.org/home/datasets), TCGA (https://portal.gdc.cancer.gov/), and GEO (https://www.ncbi.nlm.nih.gov/geo/) databases.
