# Supplementary figures and images for: A prognostic nomogram based on competing endogenous RNA network for clear‐cell renal cell carcinoma
Source: Cancer Med. 2021 Jun 30;10(16):5499–512. doi: 10.1002/cam4.4109 (PMC8366097; doi:10.1002/cam4.4109)

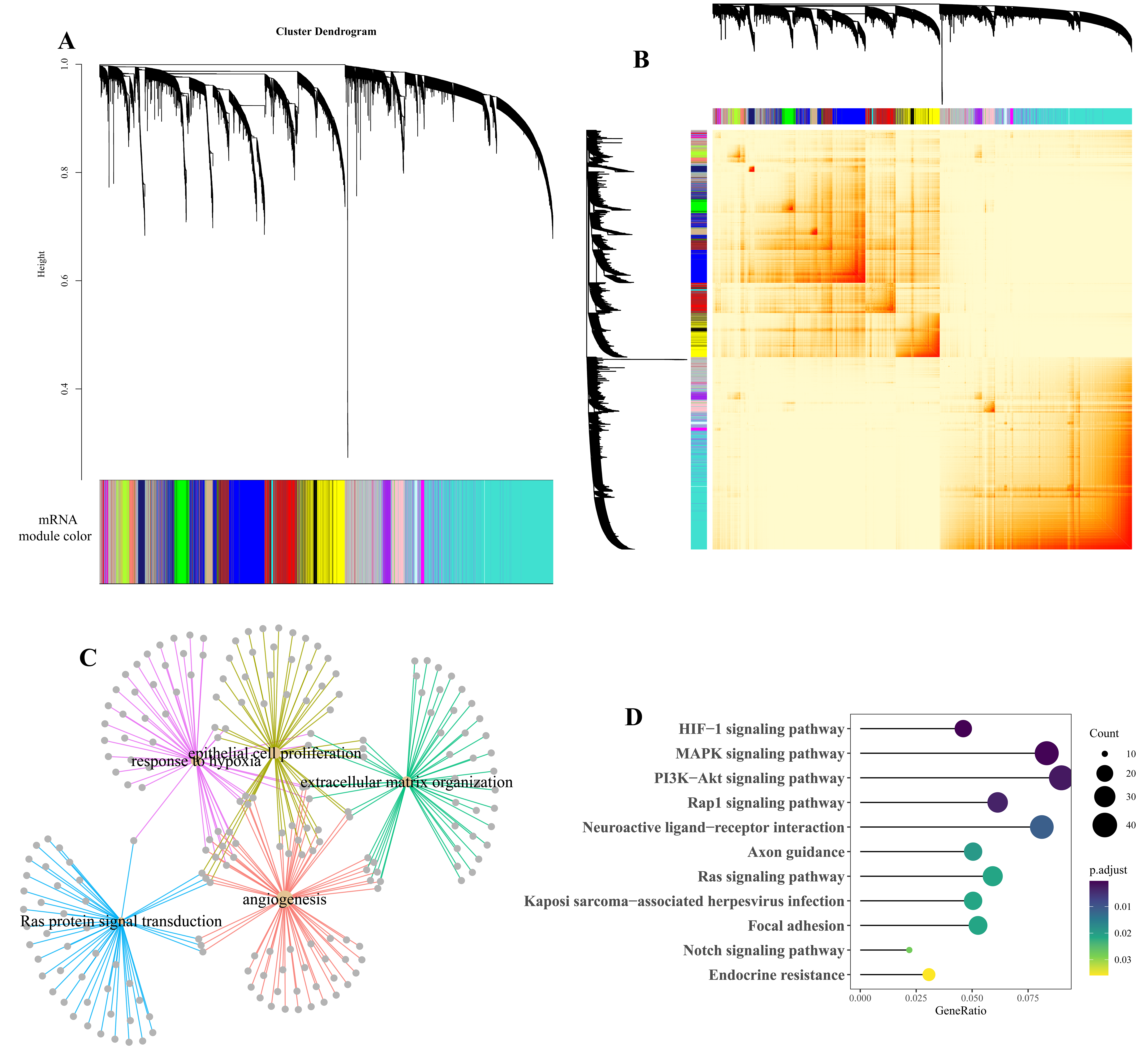

Supplement: Supplementary file 1 — Figure S1 [file CAM4-10-5499-s003.tif]

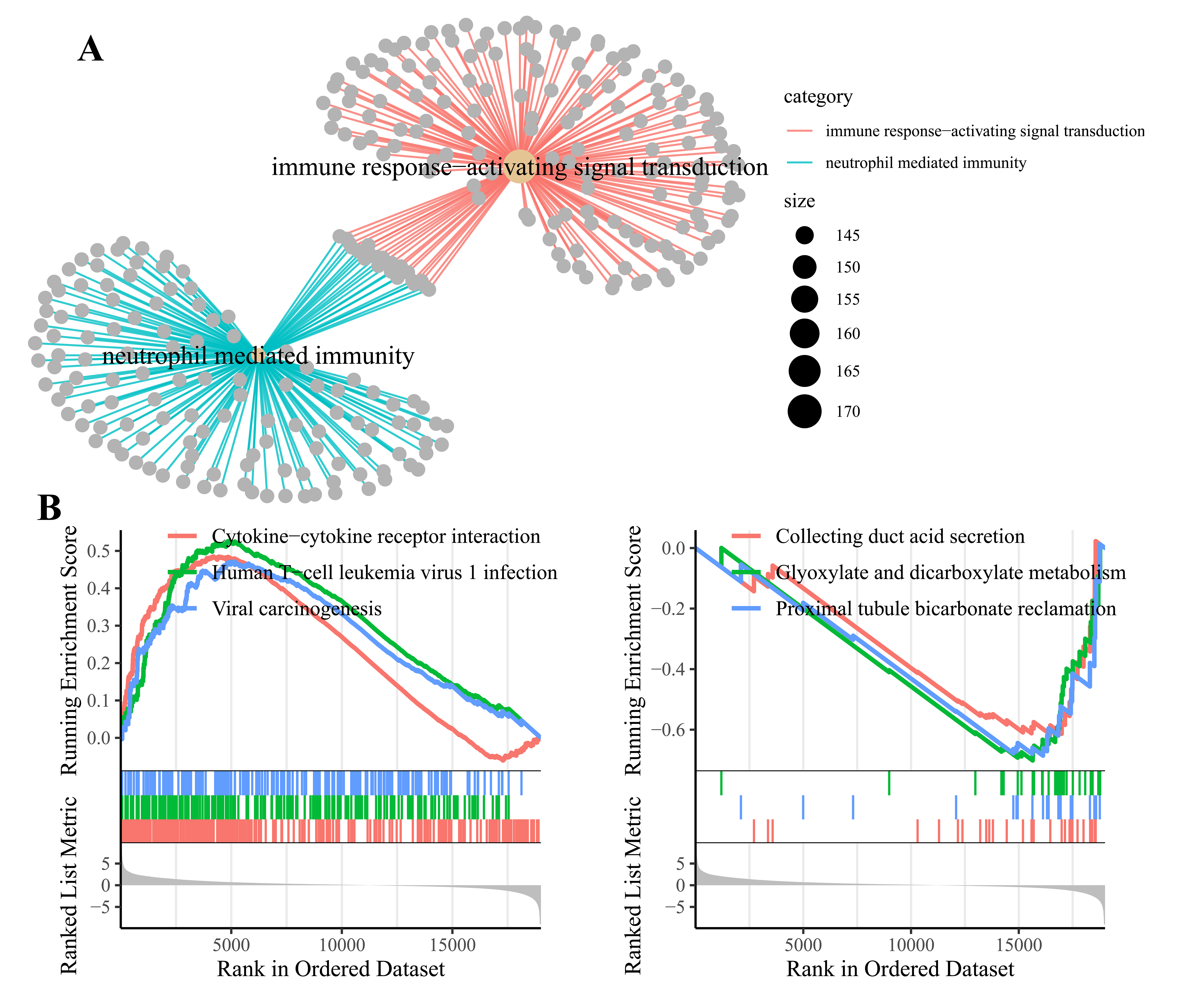

Supplement: Supplementary file 2 — Figure S2 [file CAM4-10-5499-s002.tif]

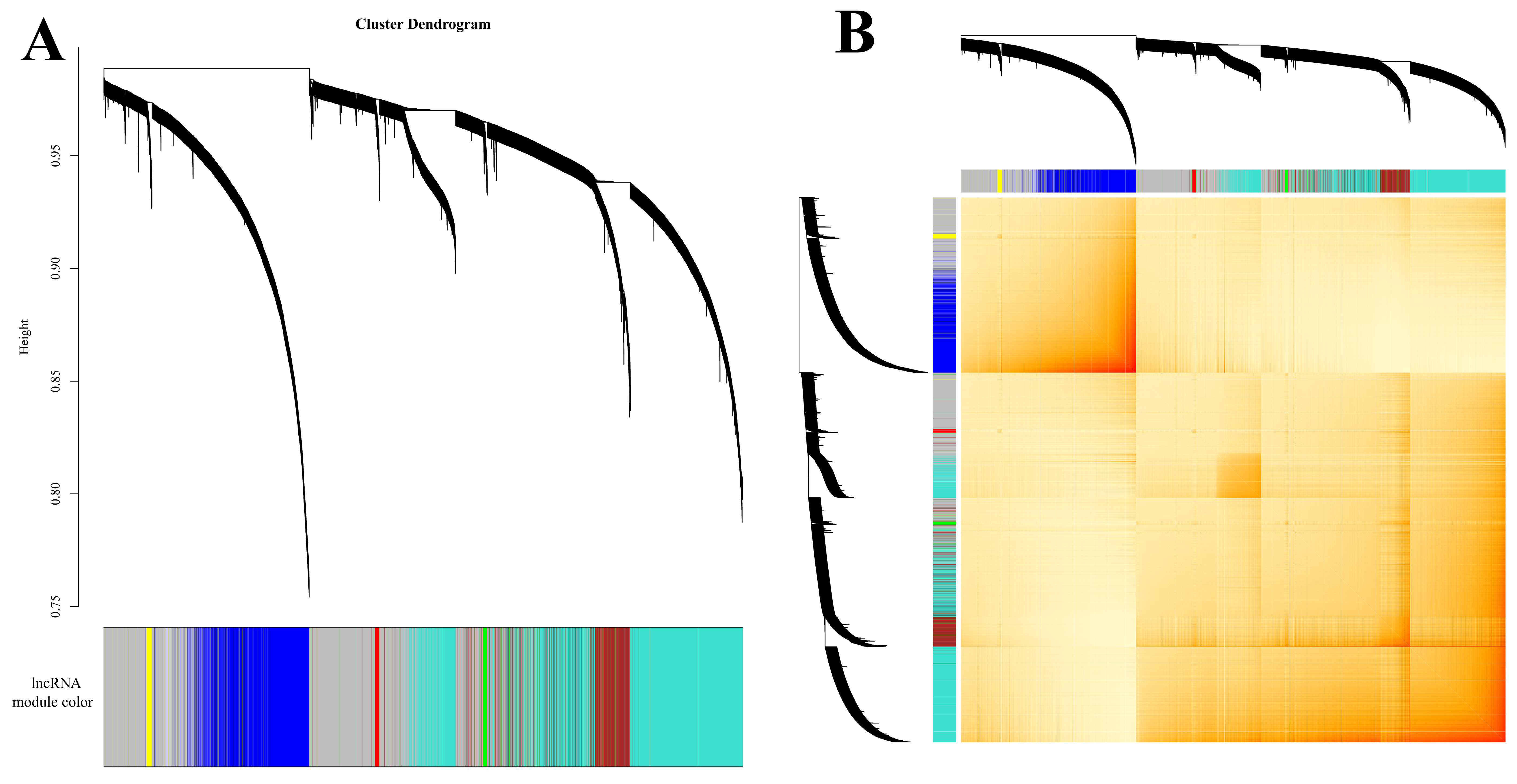

Supplement: Supplementary file 3 — Figure S3 [file CAM4-10-5499-s001.tif]
